# Associations of Circulating Insulin-Growth Factor-1 With Cognitive Functions and Quality of Life Domains in Ambulatory Young Adults With Cerebral Palsy: A Pilot Study

**DOI:** 10.3389/fneur.2022.748015

**Published:** 2022-06-27

**Authors:** Ted Kheng Siang Ng, Patricia C. Heyn, Alex Tagawa, Christina Coughlan, James J. Carollo

**Affiliations:** ^1^Department of Physical Medicine and Rehabilitation, University of Colorado Anschutz Medical Campus, Aurora, CO, United States; ^2^Edson College of Nursing and Health Innovation, Arizona State University, Tempe, AZ, United States; ^3^Center for Gait and Movement Analysis (CGMA), Children's Hospital Colorado, Aurora, CO, United States; ^4^University of Colorado Alzheimer's and Cognition Center, School of Medicine, University of Colorado Anschutz Medical Campus, Aurora, CO, United States

**Keywords:** cognition, cerebral palsy, insulin-like growth factor 1 (IGF-1), quality-of-life, patient-reported outcomes measurement information system (PROMIS), biomarkers, aging, aging model

## Abstract

**Objective:**

Adults with cerebral palsy (CP) often have impaired cognitive functions. CP also has deteriorations in multiple quality-of-life (QoL) domains. The bio-psycho-social health psychology model posits that biological factor interacts with social and psychological functions. However, the biological determinant of psycho-social and functional outcomes in CP has been scarcely examined. Circulating Insulin-like growth factor-1 (IGF-1) is associated with cognitive deficits in older adults, we thus aimed to examine the associations of circulating IGF-1 with: (1) objectively measured cognitive functions, (2) self-reported cognitive functions, and (3) QoL measures in adults diagnosed with CP.

**Methods:**

Seventy-two adults with CP and varying degrees of cognitive functions were recruited from an accredited clinical motion analysis laboratory at a regional Children's Hospital. Circulating IGF-1 was measured using post-fasting serum. The Wechsler Adult Intelligence Scale (WAIS) tests were administered to assess multiple cognitive functions, whereas the Patient-Reported Outcomes Measurement Information System (PROMIS) was used to measure multiple domains of self-reported health, including cognitive complaints and eight QoL domains.

**Results:**

Sixty-eight participants had complete data [mean age = 25 (SD = 5.3), female = 52.8%]. Controlling for covariates, circulating IGF-1 was associated with multiple cognitive domains, including positively with declarative memory and executive function and inversely with visual-spatial and motor skills, and processing speed, while no association with subjective memory complaint was detected. Circulating IGF-1 was also inversely associated with four QoL domains, including depressive symptoms, executive function, physical function, and social roles and activities.

**Conclusions:**

In CP, circulating IGF-1 might be a useful biological determinant of objective cognitive functions and several quality-of-life domains commonly impaired in CP.

## Highlights

- Circulating IGF-1 is involved in cognitive decline in normal aging and dementia. Patients with cerebral palsy frequently have co-morbid cognitive decline and thus have increased risk for developing dementia. They also have impairments in functional and quality-of-life (QoL) domains. However, whether there are associations between circulating IGF-1 and multiple neurocognitive assessments and QoL functional measures in CP is unknown.
- We showed pilot findings on circulating IGF-1 associated with multiple objective neurocognitive measures and self-reported quality-of-life outcome measures in ambulatory adults with CP.
- If validated in longitudinal causal analysis, circulating IGF-1 could potentially provide an objective and minimally invasive biomarker measure for healthcare workers, aiding risk prediction and the determination of the optimal time to introduce interventions and treatments.

## Introduction

Cerebral palsy (CP) is a lifelong condition that presents challenges at every stage of development. Evidence suggests that adults with CP are at a higher risk of developing chronic secondary health conditions associated with aging, such as diabetes, hypertension, heart diseases, and metabolic syndrome, and at a much earlier age than typically-developing adults (1–5). These chronic diseases are also risk factors for dementia and have been shown to be associated with mobility impairment, due to fatigue, walking inefficiency, frailty, and muscle and joint pain (6). Adults with CP rely on physical therapy services and novel rehabilitation research, not only to improve their day-to-day physical challenges, but also to avoid developing chronic and comorbid conditions. The health services and rehabilitation need of adults with CP are similar to the older adult population, however their health is exacerbated by CP, a pediatric onset disability, throughout their life span. Due to advancements in healthcare and specifically rehabilitation, patients with CP have longer life expectancy and often survive to old age. Despite improved longevity, compared to typically developing adults, adults with CP have impaired quality of life (QoL). The biopsychosocial model of health psychology postulates that biological factors interact and are bidirectionally influenced by social and psychological constructs, affecting physical and mental health, and dictates how people can lead healthier lives (7, 8). Thus, identifying key biological determinants, i.e., biomarkers, associated with the early deteriorations in cognitive, and functional outcomes could inform the optimal timing of implementing rehabilitation interventions for adults with CP.

One important but underdiagnosed clinical condition in adults with CP is cognitive impairment (2, 9–11). Our previous study showed that a great proportion, i.e., 75%, of our adult patients with CP screened positive for cognitive impairment defined as mild cognitive impairment (MCI) (2). We thus postulated an “accelerated aging phenomenon” for adults aging with CP, with inflammation and alterations in several vital signs posing as shared risk factors for mild cognitive impairment (MCI) and serving as plausible physiological determinants of this phenomenon (2). In our previous study, we also highlighted the need to examine insulin-growth factor 1 (IGF-1) as a potential biomarker for cognitive impairment and functions in CP (2). IGF-1 increases cell proliferation in hippocampal cells (12, 13) and its expression is also increased in the affected brain hemisphere after an ischemic injury (14, 15). Indeed, compared to matched-controls, circulating IGF-1 concentrations have been shown to be reduced in CP. A previous case-control study of fifty children and adolescents with CP were directly compared to 50 healthy age-, sex-, and pubertal stage-matched children and adolescents, showing that 62% of the participants with CP had reduced IGF-l and IGFBP-3 concentrations (16). Another study similarly showed that children with CP and retarded growth had lower circulating IGF-1 compared to healthy children controls (17). A third study showed that in 31% of patients with CP, plasma IGF-1 and IGFBP3 were under the normal values for their corresponding ages (18). Lastly and similarly, another age-matched-case-control study showed reduced serum IGF-1 in CP (19).

IGF-1 is a growth and neurotrophic hormone, that plays essential roles in body growth, tissue remodeling, neuronal plasticity, and skeletal and musculature functions (20). The receptors for IGF-1 are widely expressed in the nervous system, and IGF-1 can be regulated at the expression level and cross the blood-brain barrier (20). Despite complex roles in brain functions, IGF-1 appear to have a central role in information processing, learning and memory across the lifespan (21). Specifically, mounting evidence in the older adult suggests that reductions in circulating IGF-1 signaling are involved in cognitive decline during the normal aging process (22) and could serve as a biomarker of cognitive aging (23). Decreased concentrations of circulating IGF-1 have also been associated with, and precede, cognitive impairment in neurodegenerative conditions, such as Alzheimer's disease (AD) (22–24). Thus, low concentrations of circulating IGF-1 could serve as a risk factor for the development of AD and other types of dementia (20, 22, 24). Although we previously showed evidence supporting cognitive impairment in individuals with CP that is comparable to those observed in the older adult population with MCI (2), biological determinants of cognitive decline in adults with CP are still in scarcity (2, 25). Despite a previous study examining the roles of circulating IGF-1 in bone mineral density in young adults with CP (19, 26), no studies have associated circulating IGF-1 with cognition in adults with CP. Whether circulating IGF-1 is a biological determinant of cognitive aging in adults with CP is thus unknown. As circulating IGF-1 is a prominent biomarker for cognitive decline across multiple domains in aging, MCI, and AD (27, 28), and based on our previous findings that unveiled several commonalities between CP and MCI (2), we postulated that circulating IGF-1 could be a biological determinant and hence be associated with cognitive functions in CP.

Compared to typically-developing adults, adults with CP have worse QoL outcomes, including executive function, ambulation, and mental health (29, 30). However, whether there is a specific biological determinant which are tightly coupled with psycho-social and functional outcomes in CP has been scarcely examined. Specifically, circulating IGF-1 is a growth hormone primarily produced in the hepatocytes of the liver and, in smaller amounts, by other tissues. Due to its sensitivity to the psycho-social environment, its level can potentially reflect QoL outcomes, which encompass multiple functional and psychosocial domains. Given that circulating IGF-1 is a pleotropic biomarker associated with multiple functional and psychosocial outcomes in older populations, we postulated that circulating IGF-1 could be significantly associated with these functions in adults with CP as well.

To address these issues, iIn this study, we analyzed data from our cohort study, entitled “Cerebral Palsy Adult Transition Study (CPAT),” to evaluate circulating IGF-1 as a potential biological determinant, i.e., biomarker, for young adults with CP. The CPAT parent study's goal is to understand how young adults with CP age in general, and specifically the secondary health conditions and loss of ambulation that accompanies aging in this population (1, 2, 31–34). Specifically, in this study, capitalizing a plethora of bio-psycho-social measures, focusing on the within-population instead of between-population variability, we aimed to holistically investigate if circulating IGF-1 could be a potential biomarker associated with multiple cognitive and quality of life domains that are impaired in ambulatory young adults with CP. We thus formulated three *a priori*-set aims. The primary aim of this study is to evaluate the associations between circulating IGF-1 and multiple objective cognitive domains in CP, as assessed by the Wechsler Memory Scale-Fourth Edition (WMS-IV) and the Wechsler Adult Intelligence Scale-fourth edition (WAIS- IV) tests. Second, we focused on the Applied Cognition—General Concerns domain from the PROMIS scale as a construct for subjective cognitive concerns, and investigate its associations with circulating IGF-1. Third, we examined if circulating IGF-1 is associated with multiple quality-of-life outcomes, by employing the Patient-Reported Outcomes Measurement Information System (PROMIS) scale, which has been validated in many clinical populations, including CP (35–37). Lastly, we explored if circulating IGF-1 is significantly associated with three related biomarkers, i.e., IL-6, insulin, and IGFBP-3.

## Materials and Methods

### Study Setting and Design

This report represents data collected from the parent study, the “Cerebral Palsy Adult Transition Study” (CPAT), that had the goal of understanding how ambulatory adults with CP age. The CPAT study has been previously described (1, 2, 31). The CPAT study was performed at a regional children's hospital that has been serving the health needs of individuals with CP for over 20 years. All protocol procedures were performed under rigorous and standard clinical laboratory quality assurance. The study was approved by the University of Colorado Institutional Review Board and all participants signed informed consent prior to participation.

### Role of the Funding Source

The funders played no role in the design, conduct, or reporting of this study.

### Study Cohort and Participants

A total of 129 participants from a patient registry were identified as potential participants and underwent a short telephone screening survey to determine if they were (1) interested and able to participate in the study and (2) able to walk across a 35-foot (10.6 m) walkway, with or without assistive devices, at least three times. A total of 72 ambulatory participants were enrolled and completed the parent project (CPAT) (1, 2, 31).

**Figure 1:**
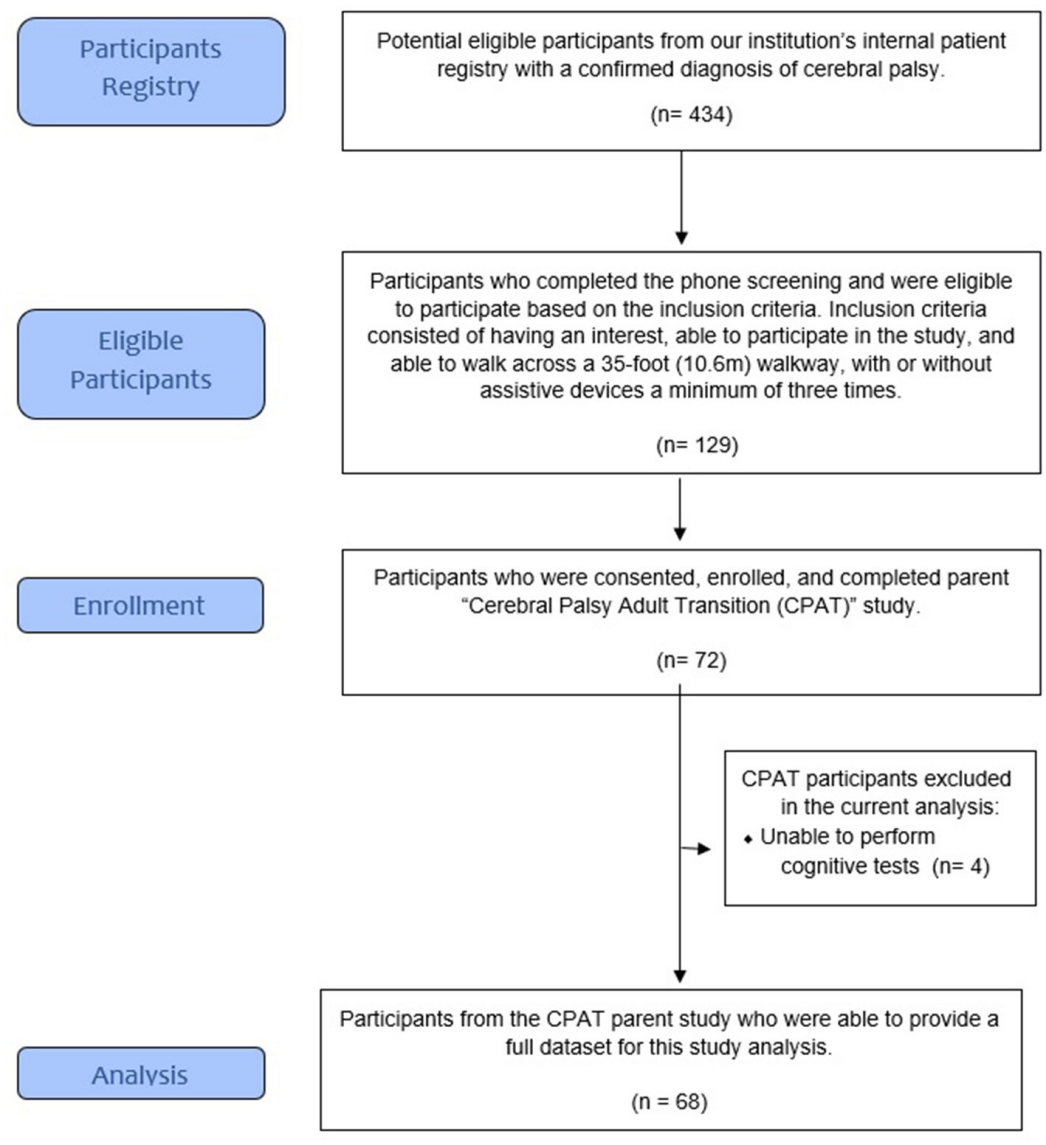
Study participant flow diagram.

For this sub-analysis, we included the data from the participants who were able to complete the cognitive and blood laboratory tests (Figure 1). All study procedures were conducted by qualified clinical and research staff utilizing an accredited, on campus, clinical laboratory who followed the institutions systematic and research training procedures.

### Assessment Tools

#### Blood Laboratory Work and Biomarker Examinations

Each participant also performed a blood draw to assess blood biomarkers. Participants fasted for 8–12 h before their blood was drawn and blood collections were scheduled in the morning to minimize diurnal variations. Blood draw *via* venipuncture was performed by a certified phlebotomist from an accredited clinical laboratory. The samples were kept at room temperature for a maximum of 3 h before being processed in the laboratory. The serum samples were then stored at −80°C until further analyses. After sample collections from all the participants were completed, all samples were assayed on the same day and on the same plates in the laboratory to avoid batch effects. Biomarker concentrations were examined in serum samples, using commercially available enzyme-linked immunosorbent assay (ELISA) kits. Four biomarkers were measured, namely IGF-1(Immunodiagnostics Systems, East Boldon, United Kingdom, USA), insulin (Immunodiagnostics Systems, East Boldon, United Kingdom), IGFBP-3 (Immunodiagnostics Systems, East Boldon, United Kingdom), and IL-6 (Meso Scale Diagnostics Maryland, USA). All the experiments were performed as per the instructions of respective manufacturers of the kits in singlets on IDS-iSYS automated instrument. The intra- and inter-assay coefficients of variations (CVs), in sequence, for the biomarkers are 2 and 9.5% for IGF-1, 1.6 and 2.8% for insulin, 3.4 and 4% for IGFBP-3, and 6 and 6.2% for IL-6.

#### Cognitive Function Assessments

To assess the detailed cognitive functions and different cognitive domains, sub-tests from the Wechsler Memory Scale IV (WMS-IV), and the Wechsler Adult Intelligence Scale IV (WAIS-IV) (39) were administered to all participants. The WMS-IV assesses a person's memory (38), while the WAIS-IV assesses cognitive ability (39). The WMS-IV assessed a person's memory, specifically a person's performance in five Index Scores: Auditory Memory, Visual Memory, Visual Working Memory, Immediate Memory, and Delayed Memory. The WAIS-IV is used as a general test the intelligence to assess overall cognitive ability for adults.

Table 2 reports all the subtests that were administered. The Short Test of Mental Status (STMS) is a screening tool for global cognitive status and was also administered during the study (40). For all three cognitive assessment tools, three researchers were trained in administering the tests and all tests were administered in the same clinical sites. The higher the scores, the better the participants' cognitive functions.

#### PROMIS-57 Scale Assessments

Patient-Reported Outcomes Measurement Information System (PROMIS)-57 was used to assess global patient-reported health-related quality of life domains, encompassing physical, mental, and social wellbeing (41). The PROMIS-57 is made up of 57 items, which assesses nine domains related to quality-of-life, including cognitive concerns, anxiety, depression, fatigue, sleep quality, participation in social roles and activities, pain interference, executive functions, and physical functions. All surveys were reviewed in person with the research team for clarity and, if necessary, were corroborated by caregivers (42, 43). For four domains, i.e., cognitive concerns, participation in social roles and activities, executive functions, and physical functions, the higher the scores, the better the participants' quality of life domains. For the other five domains, i.e., anxiety, depression, fatigue, sleep quality, and pain interference, where the higher the scores, the worse the participants' quality of life domains.

### Statistical Analyses

Based on a power calculation with 80% power at 5% significance with a 2-tailed test, a sample size of 65 could detect an effect size of 0.35 for a significant correlation. Hence, the targeted total sample size needed to be 65 or more. All continuous measures were expressed as mean ± standard deviation (SD) and categorical variables in percentages. The differences in baseline variables were examined using Student's *t*-test, chi-square or Fisher's exact tests as the data necessitated. The raw values of the biomarker measurements did not fulfill the normality assumption; therefore, they were log-transformed for subsequent analyses and were successfully normalized, based on dot plots, skewness, and kurtosis. All the analyses were performed according to the three *a priori*-set aims. In investigating aim 1, we performed linear regression analyses using the IGF-1 level as the independent variable, associating with the objective neurocognitive measures. To investigate aims 2 and 3, we used the IGF-1 as the independent variable and associated it with the different domains of the PROMIS-57 scale, which were the dependent variables. Lastly, to examine the exploratory aim, we associate IGF-1 as the independent variable with three related biomarkers, i.e., IL-6, insulin, and IGFBP-3. To control for potential confounding effects from other variables, regression analyses with and without controlling for covariates were performed; The covariates controlled for included age, sex, ethnicity, the years of education, and employment status. All the analyses were performed using the Statistical Package for the Social Sciences (SPSS) Statistics for Windows, version 24.0 (IBM Corp., Armonk, N.Y., USA). We followed other pilot studies of exploratory nature, which have adopted the practice of not correcting for multiple testing (27, 28). Hence, a two-tailed *p*-values of 0.05 and below were considered statistically significant.

## Results

### Demographics

A total of 68 participants had complete data for this study. Compared to the participants excluded from this present study, no significant differences were detected in all variables, except sex (*p* = 0.045). Table 1 summarizes the baseline characteristics of the sample. Their ages ranged from 19 to 49 years (mean = 25 years, SD = 5.35) and most participants were Caucasian (*n* = 51, 75%). Overall, the sample's sex distribution was well-balanced. The sample ambulatory characterization, measured by the Gross Motor Function Classification System I-V (GMFCS) showed that *n* = 28, 41.2% of the participants were able to walk independently (GMFCS I), *n* = 25, 36.8% were able to walk independently but with some assistance (GMFCS II), and *n* = 15, 22% were dependent of assistance and or a device to be able to ambulate (GMFCS III/IV).

**Table 1:** Demographics, clinical characteristics, and biomarker data of study participants.

**Demographics characteristics**	**CP current study** **(*N* = 68)**	**Excluded cohort participants with no neuropsychological test scores (*N* = 4)**	***P*-values**
	**Mean ±SD or *n* (%)**	**Mean ±SD or *n* (%)**	
Age (in years)	24.94 ± 5.35	25.38 ± 4.99	0.873
**Sex**			
Female	38 (55.9)	0 (0)	0.045*
Male	30 (44.1)	4 (100)	
**Ethnicity**			
Caucasian	51 (75)	4 (100)	0.566
Other ethnicities	17 (25)	0 (0)	
Years of Formal Education	13.54 ± 2.29	12.50 ± 1.00	0.370
**Employment status**			
Had active roles, either employed full time or part time, student or volunteer	44 (64.7)	1 (25)	0.290
No active roles nor employed	24 (35.3)	3 (75)	
**Biomarkers**			
Raw IGF-1; ng/mL	210.85 (72.78)	255 (68.24)	0.241
Log-transformed IGF-1	2.30 ± 0.16	2.39 ± 0.13	0.243
Raw insulin; uIU/mL	10.02 (5.79)	9.85 (5.23)	0.954
Log-transformed insulin	0.93 ± 0.26	0.93 ± 0.28	0.973
Raw IGFBP-3; ng/mL	4377.01 (929.57)	4925.50 (572.22)	0.249
Log-transformed IGFBP-3	3.63 ± 0.10	3.69 ± 0.05	0.222
Raw IL-6; pg/mL	0.53 (0.36)	0.31 (0.23)	0.223
Log-transformed IL-6	−0.38 ± 0.32	−0.61 ± 0.35	0.155
**CP Diagnosis/Subtypes**, ***N*** **(%)**			
Hemiplegic	26 (38.2)	1 (25)	1
Non-hemiplegic	42 (61.8)	3 (75)	
**STMS** ^**#**^			
Screened as having normal cognition	18 (26.5)	0 (0)	0.612
Screened as having MCI	50 (73.5)	2 (100)	
**GMFCS**, ***N*** **(%)**			
I	28 (41.2)	0 (0)	0.168
II	25 (36.8)	4 (100)	
III	13 (19.1)	0 (0)	
IV	2 (2.9)	0 (0)	
V	0 (0)	0 (0)	

STMS, Short test of mental status; GMFCS, Gross motor function classification system; #, the total n for STMS was only 70, due to two of the participants not having complete data for this measure; ^*^p < 0.05.

### Addressing Aim 1: Associations of Circulating IGF-1 With Objective Cognitive Measures

As shown in Table 2, at the bivariate level, circulating IGF-1 was only significantly associated with Total Logical Memory II—Delayed Recall Raw Score (β = 18.027, 95% CI = 2.494–33.561, *p* = 0.024). Upon controlling for covariates, circulating IGF-1 was significantly associated with multiple objectively measured and age-sensitive neurocognitive measures. They included the Total Logical Memory I—Immediate Recall Raw Score (β = 19.329, 95% CI = 2.837–35.822, *p* = 0.022), Total Logical Memory II—Delayed Recall Raw Score (β = 26.483, 95% CI = 8.885–44.082, *p* = 0.004), and Total Block Design Raw Score (β = −31.428, 95% CI = −56.325–−6.53, *p* = 0.014). Total Symbol Search Raw Score also had a trending association with circulating IGF-1 (β = −17.586, 95% CI = −35.715–0.543, *p* = 0.057).

**Table 2:** Associations between IGF-1 concentrations with objective cognitive measures.

**Objective cognitive measures**	**Models**	**IGF-1 concentrations**		
		**β (95% CI)**	***P-*values**	***R^**2**^***
Total logical memory I—immediate	Bivariate	12.821 (−1.692 to 27.333)	0.082	0.045
recall raw score	Adjusted	19.329 (2.837 to 35.822)	0.022*	0.141
Total visual paired associates	Bivariate	21.542 (−1.147 to 44.231)	0.062	0.052
I—immediate recall raw score (A + B + C + D)	Adjusted	20.5 (−6.373 to 47.373)	0.132	0.074
Total logical memory II—delayed recall	Bivariate	18.027 (2.494 to 33.561)	0.024*	0.075
raw score	Adjusted	26.483 (8.885 to 44.082)	0.004*	0.173
Total logical memory II—recognition raw	Bivariate	3.659 (−2.623 to 9.941)	0.249	0.021
score	Adjusted	4.62 (−2.41 to 11.65)	0.194	0.148
Total number of animals in 60 s	Bivariate	5.82 (−3.392 to 15.033)	0.212	0.024
	Adjusted	3.185 (−7.419 to 13.79)	0.55	0.101
Total block design raw score	Bivariate	−21.55 (−44.052 to 0.951)	0.060	0.054
	Adjusted	−31.428 (−56.325 to −6.53)	0.014*	0.196
Total digit span raw score (forward +	Bivariate	1.732 (−9.626 to 13.09)	0.762	0.001
backward + sequencing)	Adjusted	−2.025 (−14.452 to 10.403)	0.746	0.169
Total symbol search raw score	Bivariate	−6.908 (−23.501 to 9.685)	0.409	0.011
	Adjusted	−17.586 (−35.715 to 0.543)	**0.057**	0.181

IGF-1, insulin growth factor-1; 95% CI, 95% confidence interval. Bold text & * indicate trending and significant p-values, respectively. Bivariate, bivariate association between IGF-1 and cognitive domains. Adjusted, controlled for age, sex, ethnicity (Caucasian vs. Other ethnicities), years of education and employment status (active roles, either employed full time/part time, student, volunteer).

Notably, while the associations between circulating IGF-1 and Total Logical Memory I—Immediate Recall Raw Score/Total Logical Memory II—Delayed Recall Raw Score are positive, the associations between circulating IGF-1 and Total Block Design Raw Score and Total Symbol Search Raw Score were inverse. The other cognitive tests were not significantly associated with circulating IGF-1.

### Addressing Aim 2: No Associations Between Circulating IGF-1 and Subjective Cognitive Concerns

As shown in Table 3, no significant association of circulating IGF-1 with the PROMIS Applied Cognition domain, which indicated self-reported cognitive concerns, were observed at both the bivariate (β = 2.413, 95% CI = −8.689–13.514, *p* = 0.666) and multivariate concentrations (β = 3.591, 95% CI = −9.086–16.268, *p* = 0.573).

**Table 3:** Associations between IGF-1 concentrations with patient self-reported measures – PROMIS-57 scale domains.

**PROMIS-57 scale domains**	**Models**	**IGF-1 concentrations**		
		**β (95% CI)**	***P-*values**	***R^**2**^***
Applied cognition—cognitive concerns	Bivariate	2.413 (−8.689 to 13.514)	0.666	0.003
	Adjusted	3.591 (−9.086 to 16.268)	0.573	0.092
Anxiety	Bivariate	0.889 (−10.118 to 11.896)	0.873	0
	Adjusted	3.464 (−9.21 to 16.138)	0.587	0.073
Depression	Bivariate	5.873 (−3.601 to 15.347)	0.220	0.021
	Adjusted	10.642 (−0.363 to 21.647)	**0.058**	0.076
Fatigue	Bivariate	0.45 (−10.316 to 11.216)	0.934	0
	Adjusted	1.116 (−11.327 to 13.558)	0.858	0.065
Sleep disturbance	Bivariate	−2.367 (−12.097 to 7.362)	0.629	0.003
	Adjusted	−0.093 (−11.442 to 11.255)	0.987	0.052
Social roles and activities	Bivariate	−8.664 (−20.961 to 3.632)	0.164	0.028
	Adjusted	−14.397 (−28.424 to −0.369)	0.044*	0.116
Pain interference	Bivariate	−4.511 (−15.395 to 6.373)	0.411	0.010
	Adjusted	−2.629 (−15.45 to 10.191)	0.683	0.040
Executive functions	Bivariate	−9.021 (−29.228 to 11.185)	0.376	0.011
	Adjusted	−20.683 (−41.538 to 0.172)	**0.052**	0.263
Physical functions	Bivariate	−4.59 (−17.856 to 8.676)	0.492	0.007
	Adjusted	−15.234 (−29.168 to −1.3)	0.033*	0.234

IGF-1, insulin growth factor-1; 95% CI, 95% confidence interval. Bold text & * indicate trending and significant p-values, respectively. Bivariate, bivariate association between IGF-1 and PROMIS-57 Scale Domains. Adjusted, controlled for age, sex, ethnicity (Caucasian vs. Other ethnicities), years of education, and employment status (active roles, either employed full time/part time, student, volunteer).

### Addressing Aim 3: Associations of Circulating IGF-1 With Other Subjective Self-Reported PROMIS-57 Domains

As shown in Table 3, circulating IGF-1 was not significantly associated with other non-cognitive domains of PROMIS scale at the bivariate level. Upon controlling for covariates, circulating IGF-1 became significantly associated with several other non-cognitive domains of PROMIS scale, namely the PROMIS-57 Physical Function (β = −15.234, 95% CI = −29.168–−1.3, *p* = 0.033) and Social Roles & Activities sub-scales (β = −14.397, 95% CI = −28.424–−0.369, *p* = 0.044). Circulating IGF-1 also has trending associations with executive functions and depressive symptoms (β = −20.683, 95% CI = 41.538–0.172, *p* = 0.052) and (β = 10.642, 95% CI = −0.363–21.647, *p* = 0.058), respectively.

No significant nor trending association(s) were detected with the other PROMIS-57 domains, including anxiety, fatigue, sleep disturbance, and pain interference.

### Exploratory Analysis: Associations Between Circulating IGF-1 and Related Biomarkers

As shown in Table 4, circulating IGF-1 was associated significantly with IGFBP-3 (bivariate: β = 0.376, 95% CI = 0.271–0.481, *p* < 0.001& adjusted: β = 0.388, 95% CI = 0.269–0.507, *p* < 0.001) and IL-6 (bivariate: β = −0.457, 95% CI = −0.922–0.008, *p* = −0.054 & adjusted: β = −0.56, 95% CI = −1.087–−0.033, *p* = 0.038). No significant association was detected between circulating IGF-1 and insulin. No significant associations were detected between these related biomarkers with the psycho-social measures, i.e., neurocognitive assessments and PROMIS domains (data not shown).

**Table 4:** Associations between IGF-1 concentrations and other biomarkers.

**Other biomarkers**	**Models**	**IGF-1 concentrations**		
		**β (95% CI)**	***P-*values**	***R^**2**^***
Insulin	Bivariate	0.301 (−0.074 to 0.676)	0.114	0.035
	Adjusted	0.257 (−0.172 to 0.686)	0.237	0.117
IGFBP-3	Bivariate	0.376 (0.271 to 0.481)	<0.001***	0.422
	Adjusted	0.388 (0.269 to 0.507)	<0.001***	0.483
IL-6	Bivariate	−0.457 (−0.922 to 0.008)	**0.054**	0.052
	Adjusted	−0.56 (−1.087 to −0.033)	0.038*	0.146

IGF-1, insulin growth factor-1; 95% CI, 95% confidence interval. Bold text & * indicate trending and significant p-values, respectively, *** indicates p < 0.001. Bivariate, bivariate association between IGF-1 and other relevant biomarkers. Adjusted: controlled for age, sex, ethnicity (Caucasian vs. Other ethnicities), years of education, and employment status (active roles, either employed full time/part time, student, volunteer).

## Discussion

Employing the bio-psycho-social health psychology model, this is a pilot study holistically investigating IGF-1 as a biological determinant of a plethora of cognitive and QoL domains in adults with CP. Given multiple significant associations upon adjusting for pertinent covariates, IGF-1 might be a biological determinant/ biomarker of multiple objectively measured cognitive functions and subjective quality-of-life outcomes in adults with CP. These findings highlighted the pleiotropic roles of IGF-1 in aging adults with CP, particularly from the perspective of aging. On the other hand, the lack of association of IGF-1 with subjective cognitive complaint illuminated the discrepancy between objectively and subjectively measured cognitive functions, thus stressing the need to assess cognition in an objective manner in this specific population. By further showing that IGF-1 was significantly associated with and hence potentially a biological determinant of several domains of quality-of-life measures, the findings showed the pleiotropic roles of IGF-1 in young adults with CP, providing pilot data for future validation studies.

These findings illuminated the biological underpinnings of cognitive functions and suggest the utility of IGF-1 as a potential biomarker for multiple cognitive functions in young adults with CP. In particular, several of these cognitive domains include attention, memory, executive function, information processing speed, visual perception, and visuospatial skill. These functional cognitive outcomes have important implications for learning and memory, as well as daily functions, such as managing finances (44).

Considering these findings in the context of the extant literature, most studies examining the associations between IGF-1 and cognitive function in other populations have focused on global cognition (22, 27, 45). Supportive of our findings, for example, a clinical trial on children with CP has previously shown that recombinant growth hormone replacement increased IGF-1 concentrations and led to improved multiple functioning, including cognitive functions, especially psychomotor and executive functions, personal and psychosocial skills (46). Hence, IGF-1 concentrations may imply endogenous growth hormone concentrations or the liver responsiveness to growth hormone. Supporting our accelerated aging phenomenon hypothesis (2). Another study showed that IGF-1 deficient mice exhibited worsened microhemorrhages, suggesting that the pro-vascular and protective effects of circulating IGF-1 in the preservation of brain health and maintenance of cognitive function (47).

For these less-commonly-investigated specific cognitive domains, contradictory findings persist. One plausibility would be due to sensitivity issues of the neurocognitive measures used or, the biomarkers assessed in previous studies, or a combination of the two (48). For instance, a previous study showed that *lower* IGF-1 level was associated with *better* cognitive function across multiple domains (49). On the other hand, Dik et al. demonstrated that *reduced* IGF-1 predicted *declining* processing speed (48). Intriguingly, in our study, we observed a differential in the directions of effects depending on the subtype of objective cognitive measures utilized and thus the different cognitive domains. The attention and memory domains were unequivocally positively associated with IGF-1, whilst information processing speed and visual perception, and visuospatial skill were negatively associated with IGF-1 concentrations. Previous studies have shown that lower IGF-1 concentrations were associated with hippocampal atrophy (49), areas vital for learning and memory. We showed a similar trend of association, in that higher IGF-1 concentrations were associated with better learning and memory. For executive functions, a randomized controlled trial (RCT) showed that by increasing the IGF-1 concentrations in older adults, executive functioning, and to a smaller extent verbal memory, were significantly improved (50). In contrast, we showed that higher IGF-1 concentrations were associated with worse executive functions, for both objectively measured functions and those measured on a self-reported scale. One plausible reason could be that in our cohort of young adults with CP, many presented with cognitive functions comparable to MCI (2). Thus, we speculate that a physiological compensatory mechanism might be occurring, increasing IGF-concentrations above those observed in non-MCI individuals, in an attempt to buffer the neurobiological processes compromised by aging and dementia pathologies (21, 50–52). Indeed, such a compensatory mechanism of IGF-1 has been shown in upregulating the clearance mechanism of amyloid beta in the brains of older adults (53). In adults with CP, we postulate that this phenomenon could be differential, based on the hierarchy of cognitive functions, in that the compensatory mechanisms of IGF-1 kicks in at the lower hierarchy of cognition (learning and memory), and thus the positive associations. However, at the higher hierarchy of cognition (executive functions), the compensatory mechanism could have overshot and hence resulting in inverse associations with executive functions. Another interpretation could be the presence of a “threshold effect” (48), such that when IGF-1 is below a certain concentration that it is significantly associated with decline in processing speed.

Another plausible interpretation is the developmental effects of IGF-1 in cellular stress resistance and late-life mortality. A previous mice study demonstrated that a reduced IGF-1 level in the early lifespan is associated with increased lifespan, suggesting that there might be detrimental effects of high IGF-1 concentrations in the early lifespan, possibly through reduced intracerebral hemorrhage (54). In animals with significantly reduced IGF-1 concentrations caused by genetically-determined deficiencies, the incidence of other diseases, such as cancer and kidney disease, were also significantly lower. In all, while this study provided pilot and suggestive data, the contradictory roles of IGF-1 in various cognitive functions in young adults with CP warrant further investigations.

We have recently published findings similarly using the bio-psycho-social health psychology model, advocating the triaging of findings using biomarkers in older adults (55, 56). A second major finding of this study is that by demonstrating the utility of IGF-1 as an objectively-measured biological determinant, we triaged the self-reported outcomes and observed a discrepancy between objectively measured cognitive functions and self-reported cognitive complaints. These findings highlighted that cognitive decline, as measured by objective neurocognitive tests in CP, has biological underpinnings in IGF-1 in a similar manner to that observed in older adults experiencing aging, MCI and AD. On the other hand, subjective cognitive complaints, which are self-reported and subjective in nature, did not seem to a good measure for assessing cognitive health in this population, based on the lack of association with IGF-1. In contrast, subjective cognitive complaints reported by cognitively healthy older adults, have shown significant associations between the complaint scores and multiple brain degeneration markers (57–59). This discrepancy could lie in the ability of the patient to accurately appraise their own cognitive abilities, since our population had a high percentage of individuals with characteristics of MCI (75%), who could have dismissed minor cognitive issues as forgetfulness. Indeed, studies conducted with older adults with CI/MCI showed that they tend to overestimate their cognitive abilities (60). Similarly, this overestimation in young adults with CP may have attenuated the association with IGF-1. Taken together, these findings advocate that, rather than relying on quick assessment through self-reported cognitive complaints, there is likely a need for more detailed and objective assessments, either using neurocognitive tests or IGF-1, as the more accurate measures for assessing cognitive functions in young adults with CP.

The third major finding of this study is that IGF-1 was associated with four self-reported quality-of-life (QoL) domains as measured by the PROMIS scale, encompassing depressive symptoms, executive function, physical functions, and social roles and activities. Instead of divergent directions of associations as seen with objective neurocognitive measures, IGF-1 had consistent negative associations with QoL measures, in that higher IGF-1 concentrations were consistently associated with worse QoL measures. This is congruent with our postulation that IGF-1 elicits a compensatory mechanism, with higher IGF-1 concentrations compensating for deteriorating higher-level functions, such as the QoL functions. Despite presenting with contradictory findings in children with CP (46, 61), no previous studies have associated biomarker with comprehensive QoL measures in young adults with CP. Hence, this finding has potential implication in that detecting elevations in IGF-1 could be an objective measure for various declining self-reported functioning in aging adults with CP, ranging from several cognitive domains, depressive symptoms, physical functions, and social roles and activities. Importantly, these findings show that these self-reported functions are not merely subjective and fleeting feelings, and they also have a biological basis in IGF-1 concentrations, similar to how psychological wellbeing is significantly associated with IL-6 concentrations (56, 62) and metabolomics markers associated with health-related QoL measure (63). Taken together, these findings are thus supportive of the bio-psycho-social model. Hence, these findings could have profound implications in non-pharmacological interventions, especially when subjective outcome measures are the main outcome measures, with scarcity in examining objective biomarker measures. Hence, future interventional studies which focus on these self-reported and more subjective measures may be validated by concurrently measuring IGF-1.

Lastly, we showed IGF-1 was significantly associated with IGFBP-3 and IL-6. Our findings concur with those of previous studies conducted with older adults, which showed longitudinal changes in IGF-1 and IGFBP-3 were strongly and positively correlated (23). Here, for the first time, we showed the presence and the magnitudes of associations between these biomarkers in CP (45).

IGF-1 and IGFBP-3 are biomarkers of the growth hormone system, specifically insulin signaling, while IL-6 is a powerful indicator of low-grade systemic inflammation (64, 65). Our findings suggest that a network of insulin signaling dysfunction, and low-grade inflammation, potentially with alterations in IGF-1 as the precipitating event, similar to IGF-1's pleiotropic roles in other clinical conditions, partially contribute, directly or indirectly, to the objective cognitive impairments and other subjective QoL functions in young adults with CP (66, 67). With these preliminary data established and pending future validation studies, longitudinal monitoring of this panel of biomarkers could be useful in tracking subtle preclinical declines, before young adults with CP present with overt clinical symptoms, allowing for early interventions (45).

## Limitations and Strengths

We acknowledge a few limitations, such as the study's relatively modest sample size. However, to our knowledge, our cohort is the largest adult cohort with CP to date, and we extensively characterized measurements from a holistic perspective, including clinical, QoL, and biomarker measurements, representing a wide range of both objectively and subjectively measured functions in CP, which are typically not available in a single dataset. Since this is an exploratory study, we did not correct for multiple testing and, hence we cannot eliminate the possibility of having false positive findings. However, this is the first study to provide strong suggestive evidence for alterations in IGF-1 having pleiotropic roles in multiple facets of functions impaired in CP. It is worth noting that this study was of a cross-sectional design, and thus no causal relationships could be inferred from the associations, warranting longitudinal examinations.

Despite these limitations, this is the first study in the field to *a priori* examine associations of IGF-1 with multiple objective and subjective measures in adults with CP, made possible by the extensive characterizations of multimodal measures based on a holistic bio-psycho-social approach and framework. Furthermore, our regression models also controlled for several covariates, minimizing residual confounding effects. As CP is a life-long condition, but most CP studies focus solely on children with CP, this study thus addresses the scarcity of data and gaps in evidence on how patients with CP age and the risk factors associated with the aging process, which predispose them to geriatric syndromes. Upon longitudinal causal validation, these findings thus have potential clinical implications in risk prediction and interventions in this severely understudied population.

## Conclusions

Previous studies have shown that aging adults with CP suffer from secondary geriatric chronic diseases, which we termed the “accelerated aging phenomenon” (2). However, the biological determinant of this phenomenon, specifically cognitive impairment, has not been elucidated. This study filled the gaps by identifying an important biological determinant, and by extension related biological pathways, involved in multiple facets of impairments in aging adults with CP. This first insights into the underlying biological determinant of cognitive impairment and QoL functional outcomes in CP point to the potential implications of targeting a single molecule, IGF-1, as a tailored prevention and treatment strategy to maintain cognitive and physical functioning in adults with CP, along with the possibility of preventing premature secondary health conditions and cumulative impairments. Furthermore, the discrepancy observed between objectively measured cognitive functions and self-reported cognitive complaints highlights the need for detailed objective measures in this specific patient population. These findings provide the impetus for further investigations in larger studies with longitudinal design, to fully comprehend and assess the potential implications of measuring IGF-1 as a biomarker of pathology and the implications of focusing on altering IGF-1 concentrations to prevent the onset of secondary pathologies, particularly cognitive decline, observed in aging adults with CP. Lastly, gait abnormalities could manifest at early stages of Alzheimer's disease and are associated with cognitive decline, and can be used as an early biomarker to identify patients at risk of progressing to late-stage dementia. Hence, relationships between IGF-1 and cognitive tests with gait abnormalities may represent a future direction in CP research (68). These findings would have potentially important implications for cognitive impairment prevention and early intervention.

## Data Availability Statement

The data analyzed in this study is subject to the following licenses/restrictions: data could be made available on reasonable request. Requests to access these datasets should be directed to AT; Alex.Tagawa@childrenscolorado.org.

## Ethics Statement

The studies involving human participants were reviewed and approved by University of Colorado Institutional Review Board and all participants signed informed consent prior to participation. The patients/participants provided their written informed consent to participate in this study.

## Author Contributions

TN: conceptualization (present study), data analyses, data interpretation, and writing of the first draft (all sections). JC and PH: conceptualization (parent cohort). AT: data curation. TN, AT, JC, CC, and PH: methodology and critical inputs and revisions. TN, AT, and PH: project administration. TN and PH: supervision. AT and PH: intro & methods. All authors have read and agreed to the published version of the manuscript.

## Funding

This research was supported by grants from the National Institute on Disability, Independent Living, and Rehabilitation Research (NIDILRR #H133G130200, NIDILRR #90IF0055-01), in the Administration for Community Living (ACL) of the Department of Health and Human Services (HHS). Additional support was provided from the J. T. Tai & Company Foundation. The funding source was not involved in any phase of the study, including the decision to submit and publish this manuscript.

## Conflict of Interest

The authors declare that the research was conducted in the absence of any commercial or financial relationships that could be construed as a potential conflict of interest.

## Publisher's Note

All claims expressed in this article are solely those of the authors and do not necessarily represent those of their affiliated organizations, or those of the publisher, the editors and the reviewers. Any product that may be evaluated in this article, or claim that may be made by its manufacturer, is not guaranteed or endorsed by the publisher.
