# Supplementary material for: Associations of Circulating Insulin-Growth Factor-1 With Cognitive Functions and Quality of Life Domains in Ambulatory Young Adults With Cerebral Palsy: A Pilot Study
Source: Front Neurol. 2022 Jun 27;13:748015. doi: 10.3389/fneur.2022.748015 (PMC9271561; doi:10.3389/fneur.2022.748015)
Supplement: Supplementary file 1 [file Data_Sheet_1.DOCX]

**Gross motor function classification system (GMFCS) The Gross Motor Function Classification System**

(GMFCS) is a multi-level categorization tool that helps to describe varying levels of severity in people with CP [1 & 2]. The GMFCS is categorized in five different levels (I, II, III, IV, V); the lower levels (I-III) correspond with milder forms of CP, while the higher levels (IV, V) indicate increased severity. The GMFCS can be used to describe all types and severity levels of CP. This classification provides both the patient and the clinician with a description of the patient’s current motor function [2]. Typically, levels I to IV are considered ambulatory, whereas level V typically are wheelchair-bound.

**Cerebral palsy topographical classification**

The topographical classification of CP is used to diagnose and describe the body part(s) and side(s) that are affected by the condition [3]. Hemiplegia/hemiparesis is used when the arm and leg on one side of the body are affected.

**Reference**

[1]. Palisano R, Rosenbaum P, Walter S, Russell D, Wood E, Galuppi B. Development and reliability of a system to classify gross motor function in children with cerebral palsy. Developmental Medicine & Child Neurology. 1997 Apr;39(4):214-23.

[2]. Morris C, Bartlett D. Gross motor function classification system: impact and utility. Developmental medicine and child neurology. 2004 Jan;46(1):60-5.

[3]. Accardo PJ, Capute AJ, editors. Capute & Accardo's Neurodevelopmental Disabilities in Infancy and Childhood: Neurodevelopmental diagnosis and treatment. Brookes Pub; 2008.
